# StreamLight Single-Step Transepithelial Photorefractive Keratectomy (PRK) for Myopia and Myopic Astigmatism

**DOI:** 10.1155/2024/5597457

**Published:** 2024-11-14

**Authors:** David J. Gunn, Rebecca A. Cox

**Affiliations:** ^1^Department of Ophthalmology, Queensland Eye Institute, South Brisbane, Queensland, Australia; ^2^School of Medicine, The University of Queensland School of Medicine, Brisbane, Queensland, Australia

**Keywords:** photorefractive keratectomy, refractive surgery, refractive surgical procedures

## Abstract

**Background:** To report the refractive outcomes of StreamLight transepithelial photorefractive keratectomy (PRK).

**Methods:** A retrospective case series was conducted which included a total of 205 eyes of 109 patients who underwent StreamLight transepithelial PRK using the Alcon Wavelight EX500 excimer laser. All eyes had myopia or myopic astigmatism, and the preoperative spherical equivalent (SEQ) ranged from −0.63D to −7.25D. The primary postoperative outcomes were UDVA, CDVA and subjective refraction measured at least 3 months postoperatively.

**Results:** Postoperatively, 196 eyes (95.6%) had a UDVA of 20/20 or better. The mean SEQ was −0.05 ± 0.31D and 189 eyes (92.2%) were within ±0.50D of the target SEQ. The mean refractive astigmatism was −0.28 ± 0.27D, and 181 eyes (88.3%) had ≤ 0.50D of astigmatism. The mean safety and efficacy indices were 1.01 ± 0.08 and 0.97 ± 0.12, respectively. Eight eyes lost 1 line of CDVA. Six of these were noted to have significant dry eyes and 2 had corneal haze. No eye lost two or more lines of CDVA.

**Conclusions:** StreamLight transepithelial PRK results in excellent refractive outcomes for myopia and myopic astigmatism.

## 1. Introduction

Photorefractive keratectomy (PRK) was first introduced as a method for myopic refractive correction over 3 decades ago [1]. However, in the 1990s the popularity of PRK declined following the emergence of laser-assisted in situ keratomileusis (LASIK), which offered reduced postoperative pain and a faster visual recovery compared to PRK [2]. Nevertheless, PRK remains a safer option for laser refractive surgery in patients with thin or irregular corneas due to the greater alteration of corneal biomechanical strength and higher risk of ectasia following LASIK [3, 4]. PRK also has the advantage of reduced postoperative dry eye compared to LASIK, and avoids intraoperative and postoperative flap complications [2, 5].

In an attempt to reduce the postoperative pain and corneal haze associated with conventional PRK, various alternative methods of epithelium removal have been described, including transepithelial PRK, in which the epithelium is removed using excimer laser ablation. Early attempts at transepithelial PRK used a two-step method, which required reprogramming of the laser platform between the epithelial removal phase and the treatment phase [6]. This was associated with corneal dehydration and consequently the outcomes of 2-step transepithelial PRK are not superior to conventional PRK [6–8]. More recently, single-step transepithelial PRK has been described in which the epithelial removal and stromal ablation are performed using a single step, minimising corneal dehydration [9].

Studies investigating the outcomes of single-step transepithelial PRK, including two studies using StreamLight [10, 11], have confirmed that this modification on conventional PRK results in similar efficacy compared to both alcohol-assisted and mechanical PRK [9, 12–15]. In addition, a recent meta-analysis found no difference in the safety, efficacy, and predictability between transepithelial PRK, conventional PRK, laser epithelial keratomileusis (LASEK), and epi-LASIK [16], highlighting it as a viable alternative for refractive laser surgery. Transepithelial PRK has also been shown to result in reduced higher order aberrations compared to both LASIK and small incision lenticule extraction (SMILE) [17, 18].

One challenge with transepithelial PRK is achieving accurate epithelial ablation across the entire treatment zone due to the reduced energy of the obliquely incident laser rays reaching the peripheral corneal surface [19], and the variation in epithelial thickness across the cornea [20]. Because of this, early studies of transepithelial PRK reported shallow peripheral ablation and epithelial remnants resulting in increased ablation of the central zone and subsequent over-corrections [6, 8, 21, 22].

Currently, there are a number of commercially available platforms capable of performing single-step transepithelial PRK; the Amaris 500, Amaris 750S, and Amaris 1050RS (SCHWIND eye-tech-solutions), the iVis laser suite (iVis Technologies), and the Wavelight EX500 (Alcon Laboratories), and while there are numerous studies investigating the long-term outcomes of the Amaris 500, there has been comparatively little research into outcomes of transepithelial PRK using the Wavelight EX500 [10, 11, 23–33]. This study reports the spherical and astigmatic refractive outcomes of eyes treated using the Alcon Wavelight EX500 StreamLight technology, a single-step transepithelial PRK procedure which utilises an optimised epithelial ablation profile and allows for customisation of the epithelial ablation depth according to the individual central corneal epithelial thickness to ensure accurate treatment across the entire ablation zone.

## 2. Methods

All patients who underwent StreamLight transepithelial PRK between January 2020 and August 2023 at South Bank Day Hospital, Brisbane, Australia, were retrospectively enrolled into the study. Eyes were excluded if there was concurrent ocular disease, a history of previous refractive laser surgery, multifocal intraocular lenses, amblyopia, irregular corneal topography, preoperative corrected distance visual acuity (CDVA) worse than 0.0 logMAR, a monovision refractive target, hyperopic refractive error, if PRK was performed in combination with collagen-cross linking, or if the patient was lost to follow-up before 3 months. This study was performed in accordance with the tenets of the Declaration of Helsinki and was approved by the Royal Australian and New Zealand College of Ophthalmologists Human Research and Ethics Committee. Informed written consent was obtained from all patients. This study was supported by an Investigator-Initiated Trial grant from Alcon Laboratories.

A comprehensive preoperative assessment was performed on each patient including age, gender, racial background, CDVA, autorefraction (Nidek AR-1a, Nidek Co. Ltd.), subjective refraction, rebound tonometry (Icare, Tiolat Oy), slit lamp examination and fundus assessment. Ancillary testing included corneal tomography (Pentacam HD, Oculus), corneal biomechanics (Corvis ST, Oculus), and epithelial thickness mapping (Cirrus 5000 OCT, Zeiss). Soft contact lens wear was discontinued for a minimum of 7 days prior to the preoperative assessment.

All surgery was performed by a single surgeon at one site, using the Wavelight EX500 excimer laser (Alcon Laboratories). The Alcon Wavelight Wavefront Optimised Profile Nomogram was applied for all StreamLight treatments and was not modified.

Patients were offered oral diazepam 5 mg 30 min before surgery, and a single drop of preservative-free topical anaesthetic was instilled three times in the 15 min before surgery. Periocular povidone iodine 5% was applied to the eyes and a Lieberman eye speculum was placed. The ocular surface sensation and appearance were assessed under the excimer microscope. If sensation was present, another drop of topical anaesthetic was placed. 10 mL of chilled balanced salt solution (BSS) was irrigated to cool and clean the corneal surface. Moistened nonshedding polyvinyl alcohol spears we used to dry the cornea.

Single-step transepithelial ablation was performed with a 10 s interval between the epithelial and refractive ablation and all attempts were made to complete treatment segments without any other pauses. Pupil tracking was activated for all treatments with no re-centration required between modes. Cyclotorsion control was utilised when available. The epithelial ablation diameter was set to 0.5 mm larger than the treatment diameter. The central epithelial ablation depth was set to be at least 3 *μ*m more than the patient's OCT measured central epithelial thickness to ensure complete ablation of the epithelium across the entire treatment zone.

Following completion of excimer treatment, a PVA corneal light shield soaked in mitomycin C 0.02% was placed on the stromal bed for 20 s in all patients. The ocular surface was then immediately irrigated with at least 5 mL of chilled BSS, followed by instillation of four drops of tobramycin 0.3%. A bandage contact lens was then placed (PureVision 2, Bausch and Lomb), which had been presoaked in preservative-free ketorolac tromethamine 0.45% for at least 30 min. This was removed at the first visit 5–7 days after surgery.

Postoperative topical medications included dexamethasone eye drops 0.1% four times per day for 21 days, tobramycin eye drops 0.3% six times per day for 7 days, and preservative free artificial tears four times per day for 2 months. Oral paracetamol with ibuprofen (500 mg/150 mg) four times per day and oxycodone with naloxone (10 mg/5 mg) two times per day were prescribed for pain management if needed. Each patient was provided with and strongly advised to wear UV protection wraparound sunglasses whenever outdoors for 3 weeks.

Follow up assessments were performed at 1 week, 1 month, 3 months and 12 months. At each follow up visit, UDVA was measured. Subjective refraction and CDVA were also measured at the 1, 3 and 12-month follow up visits.

All statistical analyses were performed using SPSS version 29.0 (SPSS Inc., IBM). Continuous variables are presented as the mean ± standard deviation (SD) and analysed using independent samples *t* tests. Categorical variables are presented as frequency and percentage and analysed using Chi-square or Fisher exact tests. A *p* value of < 0.05 was considered significant.

To investigate the astigmatic correction achieved, vector analysis was performed using the Alpins method [34] to determine the target-induced astigmatism (TIA) vector and the surgically induced astigmatism (SIA) vector. The magnitude of error and the angle of error were defined as the difference between the TIA and SIA vectors. A positive angle of error represents counter clockwise error, and a negative angle of error represents clockwise error. The safety index was defined as the postoperative CDVA divided by the preoperative CDVA, and the efficacy index as the postoperative UDVA divided by the preoperative CDVA. The spherical equivalent (SEQ) correction index is defined as the ratio of the achieved SEQ to the attempted SEQ, and the astigmatic correction ratio as the ratio of the SIA magnitude to the TIA magnitude. Standard reporting graphs for refractive surgery are presented [35].

## 3. Results

A total of 205 eyes of 109 patients were included in the study. The mean age of patients was 32.5 ± 6.0 years (range: 21.5–54.3 years), and 68 (62.4%) were female. Preoperative and postoperative data are presented in Table 1. The majority of eyes (72.0%) had myopic astigmatism, 23 eyes (11.2%) had simple myopia, and two eyes (1.0%) had simple myopic astigmatism. Eight eyes (3.9%) were from participants of Asian descent.

The surgical parameters for all eyes are shown in Table 2, including the total surgical time, break time between the epithelial and stromal ablation, and the maximum ablation depth. The break time between epithelial removal and ablation was between 9 and 12 s for 185 eyes (90.2%), 13–19 s for 14 eyes (6.9%), 22–50 s for five eyes (2.4%) and one eye had a break time of 104 s. There was no significant correlation between break time and postoperative SEQ. The average time between surgery and the most recent postoperative visit was 10 ± 5 months, and 162 eyes had a postoperative period of 6 months or more.

**Figure 1:**
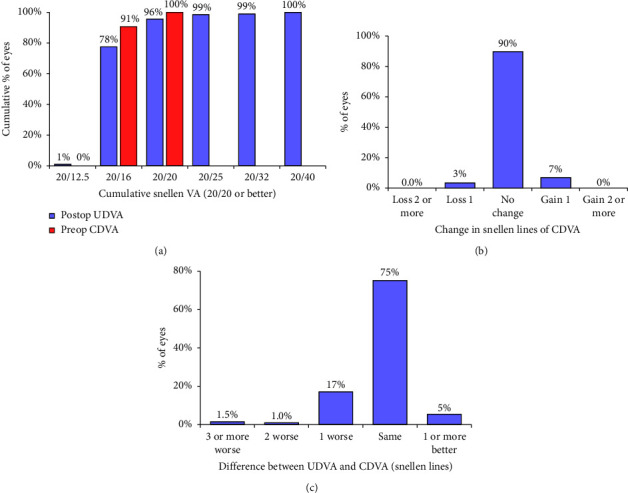
Standard graphs for reporting refractive surgery outcomes including (a) cumulative histogram of preoperative CDVA and postoperative UDVA, (b) the change in CDVA from the preoperative to postoperative assessment and (c) the difference between preoperative CDVA and postoperative UDVA.

The mean UDVA was −0.1 ± 0.1 logMAR (range: −0.2 to 0.3 logMAR) and 196 eyes (95.6%) had a UDVA of 20/20 or better (Figure 1(a)). Analysis of the preoperative and postoperative CDVA showed good safety of the procedure with only seven eyes (3.4%) losing 1 line in CDVA (Figure 1(b)). The cause of this was identified as dry eyes (5 eyes), and mild corneal haze (2 eyes). No eye lost two or more lines of CDVA. Fourteen eyes (6.8%) gained 1 line in CDVA. The mean safety index was 1.01 ± 0.08. In regards to efficacy of the procedure, 165 eyes (80.5%) had a postop UDVA that was the same or better than their preoperative CDVA, 35 eyes (17.1%) lost 1 line (of these 31 were 20/20 postop and 4 were 20/25), 2 eyes (1.0%) lost 2 lines and three eyes (1.5%) lost three or 4 lines (one of which had significant dry eyes, one had corneal haze, and one had an over-correction) (Figure 1(c)). The mean efficacy index was 0.97 ± 0.12.

**Figure 2:**
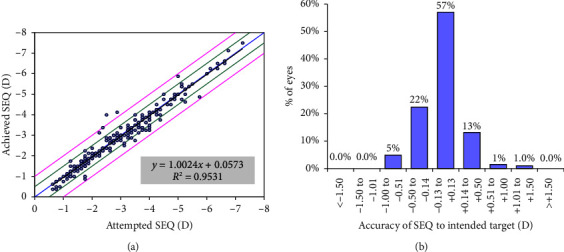
Standardised graphs for reporting refractive surgery outcomes including (a) regression analysis of the attempted versus the achieved spherical equivalent (SEQ), the black line indicates the regression line and (b) the accuracy of the SEQ to the intended target.

Linear regression analysis of the attempted versus achieved SEQ showed excellent accuracy of the procedure with a regression slope of 1.0024 (Figure 2(a)). The mean SEQ was −0.05 ± 0.31D and 189 eyes (92.2%) were within ± 0.50D of the target SEQ (Figure 2(b)).

**Figure 3:**
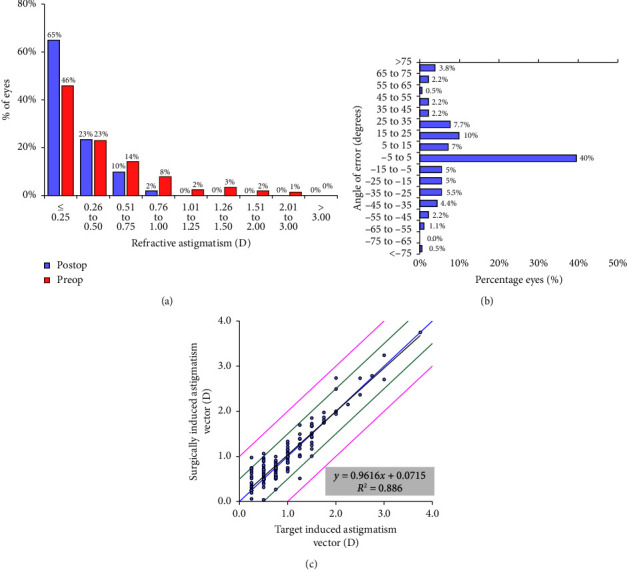
Standardised graphs for reporting refractive surgery outcomes including (a) the distribution of preoperative and postoperative refractive astigmatism, (b) the angle of error of postoperative astigmatism and (c) linear regression analysis of the target-induced astigmatism (TIA) vector versus the surgically-induced astigmatism (SIA) vector, the black line indicates the regression line.

The mean refractive astigmatism was −0.29 ± 0.27D and 181 eyes (88.3%) had ≤ 0.50D of refractive astigmatism (Figure 3(a)). The angle or error was within ±15° for 95 eyes (52.2%) (Figure 3(b)). As illustrated in Figure 3(c), the astigmatic correction also achieved good accuracy with a TIA versus SIA regression slope of 0.96. The mean astigmatic correction index was 1.12 ± 0.48.

Table 3 shows the comparisons in the safety, efficacy and refractive predictability between low myopia (≤3.00D) and moderate myopia (>3.0D), and low astigmatism (<1.50D) and moderate astigmatism (≥1.50D). There was no significant difference in the safety, efficacy, or refractive predictability between low and moderate myopia. However, eyes with low astigmatism (<1.5D) had a lower mean residual astigmatism (*p*=0.003) and a mean efficacy index closer to one (*p*=0.02), compared to eyes with moderate astigmatism (≥1.50D). The correction index of eyes with low astigmatism was significantly greater than that of eyes with moderate astigmatism (*p*=0.011).

## 4. Discussion

This retrospective study reports the refractive outcomes of StreamLight transepithelial PRK, and shows that this procedure provides excellent outcomes with good refractive predictability in eyes with primarily low to moderate myopia and myopic astigmatism. This study adds to the small amount of existing literature which has previously reported on the outcomes of StreamLight PRK, most of which have included a small sample size of 50 eyes or less [11, 23–25, 28, 30].

The potential benefits of single-step transepithelial PRK over the two-step method are the reduced treatment time and shorter break time between modes leading to reduced corneal dehydration and improved predictability. While some studies comparing different PRK methods have reported that StreamLight results in faster epithelial healing and reduced postoperative pain [10, 25], others have reported greater pain on day 1 [23, 27]. Given that postoperative pain is one of the greatest drawbacks of PRK, more research in this area is needed.

In the current study, both the safety and efficacy indices were excellent, with outcomes comparable to other methods of transepithelial PRK [15, 21, 22, 36], conventional PRK [16], LASIK [37, 38], LASEK and epi-LASIK performed on normal corneas [16]. This study found comparable results to the previous study of StreamLight by Abdelwahab, Salem and Elfayoumi [25] in regards to the proportion of eyes that achieved a UDVA of 20/20 or better (96% vs. 98%), and the proportion of eyes that were SEQ within ± 0.50D of the target SEQ (92% vs. 91%). However, a slightly smaller proportion of eyes in the current study were within 0.50D of the target astigmatic correction (88% vs. 94%).

A total of 7 eyes lost one line of CDVA, of which 5 had significant dry eyes and were undergoing treatment including topical lubricants and intense pulsed light therapy. Significant corneal haze was noted in the other two eyes, both of which were treated with topical steroids and lubricants. Comparatively, previous studies of StreamLight have reported visually significant haze in 0%-1% of eyes [24–26, 28, 29], while in studies using the Amaris 500 platform, haze occurrence ranges from 2%–9% [15, 39–42]. Similarly, the Amaris 750S and 1050RS have reported haze rates of 6% and 3%, respectively [43–45]. All of these studies, including the current study, used intraoperative 0.02% Mitomycin C which has been shown to reduce postoperative haze [46], however, comparisons should be made with caution given the number of variables which can impact haze occurrence. The concentration and application time of Mitomycin C was kept consistent for all eyes to remove the potential impact on early refractive outcomes. One limitation of this study is that no grading scale was used for corneal haze. Additionally, measurement of epithelial healing time and postoperative pain were outside the scope of the current study.

We found no significant difference in the safety and efficacy indices or in the refractive predictability between eyes with mild and moderate myopia. However, compared to eyes with low astigmatism, those with moderate astigmatism had a greater degree of residual astigmatism. Although the difference was statistically significant, the magnitude of the difference was less than 0.25D and therefore unlikely to be of clinical significance. In addition, vector analysis revealed no significant difference in the magnitude or the angle of astigmatic error between these two groups. There was a slight tendency for a low degree of overcorrection of astigmatism for those with low astigmatism with a correction index of 1.15 ± 0.52.

For the spherical correction, the regression slope of attempted v achieved SEQ was close to 1, with no significant trend for over or under correction. While some studies have suggested that transepithelial PRK results in overcorrected SEQ compared to conventional PRK methods [21, 47], this was not observed in to be the case for StreamLight transepithelial PRK, using the appropriate nomogram.

Transepithelial PRK is an excellent option for laser refractive surgery. It removes the need for alcohol and manipulation of the surface of the cornea and previous studies have indicated that it potentially offers reduced postoperative pain and faster re-epithelialisation compared to conventional methods. In the current study, StreamLight single-step transepithelial PRK has shown excellent results for the correction of low to moderate myopia and myopic astigmatism. Future studies should investigate the outcomes of StreamLight for treating hyperopia and mixed astigmatism which has not yet been done.

## Figures and Tables

**Table 1:** Preoperative and postoperative patient characteristics for 205 eyes.

Parameter	Mean ± SD	Range	*p* value
SEQ (D)^a^			
Preoperative	−3.01 ± 1.40	−7.25 to −0.63	< 0.001
Postoperative	−0.05 ± 0.31	−0.88 to 1.50	
Astigmatism (D)			
Preoperative	−0.77 ± 0.63	−3.75 to 0.00	< 0.001
Postoperative	−0.29 ± 0.27	−1.00 to 0.00	
Flat Kb (D)			
Preoperative	44.1 ± 1.5	40.1 to 47.3	< 0.001
Postoperative	40.5 ± 1.8	36.2 to 45.2	
Steep K (D)			
Preoperative	43.1 ± 1.5	39.3 to 47.0	< 0.001
Postoperative	41.2 ± 1.8	36.9 to 45.9	
CCTc (*μ*m)			
Preoperative	544 ± 31	457 to 641	< 0.001
Postoperative	497 ± 38	409 to 600	
CDVAd (logMAR)			
Preoperative	−0.09 ± 0.03	−0.10 to 0.00	0.13
Postoperative	−0.09 ± 0.03	−0.20 to 0.00	

^a^Spherical equivalent.

^b^Keratometry.

^c^Central corneal thickness.

^d^Corrected distance visual acuity.

**Table 2:** Surgical parameters for all included eyes (*n* = 205).

Surgical parameter	Mean ± SD	Minimum	Maximum
Total time (seconds)	44 ± 10	16	139
Break time (seconds)	12 ± 8	9	104
Maximum ablation (*μ*m)	52.43 ± 18.96	15.53	104.75

**Table 3:** Comparison of safety and efficacy outcomes between low and moderate myopia, and low and moderate astigmatism.

Outcome	Low myopia ≤ 3.0D (*n* = 129)	Moderate myopia > 3.0 to 6.0D (*n* = 76)	*p* value	Low astigmatism < 1.5D (*n* = 174)	Moderate astigmatism ≥ 1.5D (*n* = 31)	*p* value
Within ± 0.50D (%)	121 (93.8%)	68 (89.5%)	0.27	157 (90.2%)	24 (77.4%)	0.063
Mean SEQ/astigmatism (D)	−0.02 ± 0.32	−0.10 ± 0.30	0.09	−0.26 ± 0.26	−0.41 ± 0.28	0.003^∗^
Mean safety index	1.00 ± 0.07	1.02 ± 0.09	0.12	1.01 ± 0.07	0.99 ± 0.08	0.09
Mean efficacy index	0.96 ± 0.12	0.97 ± 0.13	0.54	0.98 ± 0.11	0.91 ± 0.15	0.02^∗^
Mean correction index	0.98 ± 0.16	0.97 ± 0.07	0.91	1.15 ± 0.52	1.02 ± 0.14	0.011^∗^
Magnitude of astigmatic error (D)	0.03 ± 0.18	0.05 ± 0.25	0.69	0.04 ± 0.20	0.04 ± 0.24	0.89
Absolute angle of error (degrees)	3.46 ± 26.69	7.06 ± 33.54	0.45	4.93 ± 31.67	4.19 ± 14.15	0.84

^∗^Significant at the level of < 0.05.

## Data Availability

The data that support the findings of this study are available from the corresponding author upon reasonable request.
